# Different Antifungal Activity of *Anabaena* sp., *Ecklonia* sp., and *Jania* sp. against *Botrytis cinerea*

**DOI:** 10.3390/md17050299

**Published:** 2019-05-20

**Authors:** Hillary Righini, Elena Baraldi, Yolanda García Fernández, Antera Martel Quintana, Roberta Roberti

**Affiliations:** 1 Department of Agriculture and Food Sciences, Alma Mater Studiorum, University of Bologna, 40127 Bologna, Italy; hillary.righini2@unibo.it (H.R.); elena.baraldi@unibo.it (E.B.); 2 Banco Español de Algas, Instituto de Oceanografía y Cambio Global, IOCAG, Universidad de Las Palmas de Gran Canaria, 35214 Telde, Las Palmas, Canary Islands, Spain; garcia.fernandez.yolanda@gmail.com (Y.G.F.); amartel@marinebiotechnology.org (A.M.Q.)

**Keywords:** antifungal activity, polysaccharides, algae, cyanobacteria, *Botrytis cinerea*, strawberry

## Abstract

Water extracts and polysaccharides from *Anabaena* sp., *Ecklonia* sp., and *Jania* sp. were tested for their activity against the fungal plant pathogen *Botrytis cinerea*. Water extracts at 2.5, 5.0, and 10.0 mg/mL inhibited *B. cinerea* growth in vitro. Antifungal activity of polysaccharides obtained by *N*-cetylpyridinium bromide precipitation in water extracts was evaluated in vitro and in vitro at 0.5, 2.0, and 3.5 mg/mL. These concentrations were tested against fungal colony growth, spore germination, colony forming units (CFUs), CFU growth, and on strawberry fruits against *B. cinerea* infection with pre- and post-harvest application. In in vitro experiments, polysaccharides from *Anabaena* sp. and from *Ecklonia* sp. inhibited *B. cinerea* colony growth, CFUs, and CFU growth, while those extracted from *Jania* sp. reduced only the pathogen spore germination. In in vitro experiments, all concentrations of polysaccharides from *Anabaena* sp., *Ecklonia* sp., and *Jania* sp. reduced both the strawberry fruits infected area and the pathogen sporulation in the pre-harvest treatment, suggesting that they might be good candidates as preventive products in crop protection.

## 1. Introduction

*Botrytis cinerea* is the causal agent of grey mold and it is among the most important postharvest fungal pathogens worldwide. This fungus can infect a large host range of fruits such as pea, kiwi, grape berry, golden berry, tomato, and strawberry [1,2,3,4,5,6].

The control of fungal diseases is usually based on synthetic pesticides; however, the European Directive 2009/128/EC on implementation of bio-based strategies supports the development of sustainable agriculture protection management through different means, among them natural products. Extracts obtained from algae and cyanobacteria may be considered a useful tool for disease control. Indeed, algae and cyanobacteria extracts showed activity against several plant pathogens [7,8,9,10,11,12,13]. Recent studies demonstrated that extracts from the two brown algae *Laminaria digitata* and *Undaria pinnatifida* and from the red one *Porphyra umbilicalis* inhibited both *B. cinerea* mycelial growth and spore germination [14]. Another extract from the brown alga *Lessonia trabeculata* displayed a protective effect against *B. cinerea* on tomato leaves [11]. Moreover, in horticultural plants algal and cyanobacterial extracts were capable of increasing the transcription and the activity of defense-related enzymes involved in the control of fungal pathogens [9,15,16,17].

Few studies have examined the antifungal activity of the single compounds contained in the extracts such as polysaccharides, phenols, and cyclic peptides [18,19]. In particular, sulfated polysaccharides, such as carrageenan, fucoidan, and ulvan showed antimicrobial activity against human pathogens [20,21,22]. The bioactivity degree of these compounds might be related to their structure, molecular size, and sulfate groups amount [22,23]. Moreover, polysaccharides played a role as inducers of plant resistance, since they increased the activity of various defense-related enzymes such as chitinase, β-1,3-glucanase, peroxidase, polyphenol oxidase, phenylalanine ammonia lyase, and lipoxygenase [8,19]. Ulvans are the principal polysaccharides contained in the cell walls of green algae, whereas agarans and carrageenans are the principal polysaccharides for red algae and alginates and fucans for brown algae [19,24]. Species of brown alga *Laminaria* are sources of laminarin, a storage polysaccharide exploited in plant protection, already commercialized in many EU countries (EU Pesticide Database) for its capacity to induce plant resistance. Among phenols, bis (2,3-dibromo-4,5-dihydroxybenzyl) ether displayed antifungal activity of *B. cinerea* growth and decreased the incidence of fruit decay and disease severity of strawberry fruits infected with the pathogen [25]. On the other hand, to our knowledge, no studies have been carried out on the antifungal activity of cyanobacterial polysaccharides against plant pathogens, and also their activity against human pathogens is poorly documented [26,27].

On the basis of these considerations, the objectives of the present work were to study the antifungal activity of (i) extracts from *Anabaena* sp., *Ecklonia* sp., and *Jania* sp. against *B. cinerea* colony growth; (ii) polysaccharides extracted from the extracts against pathogen fungal growth, spore germination, colony forming units; (iii) polysaccharides applied by pre- or post-harvest treatment against grey mold disease on strawberry fruits under greenhouse conditions.

## 2. Results

### 2.1. Antifungal Activity of Water Extract (WE) and Polysaccharides (POL)

The colony growth rate of *B. cinerea* in presence of different WE treatment and concentrations was calculated (Table 1). Two-way ANOVA indicated that WE treatment and WE concentration factors were significant (*p* < 0.05), whereas the interaction between the two factors was not significant. In particular, *B. cinerea* growth was inhibited by all the WE treatments in a similar way. WE had an effect on fungal growth directly depending on the concentration, ranging from 6% to 9.8% for 2.5 and 5 mg/mL, respectively. No further significant increase in the inhibitory effect was detected if the concentration increased above 5 mg/mL.

**Figure 1:**
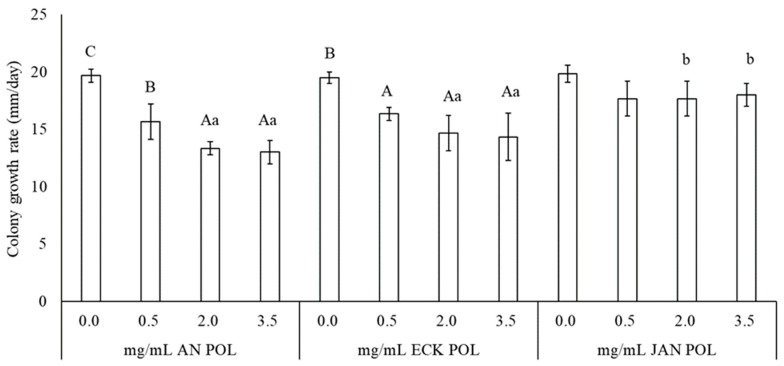
Effect of different POL concentrations on *Botrytis cinerea* colony growth rate. Treatment and dose factors and their interaction are significant, according to factorial ANOVA. F_(2,36)_ = 18.2, *p* < 0.0001 (for treatment factor), F_(3,36)_ = 27.8, *p* < 0.0001 (for dose factor), F_(6,36)_ = 2.7, *p* < 0.05 (for interaction). Columns are mean values ± SD. The same uppercase letter within each POL treatment and the same lowercase letter among concentrations indicates no significant differences according to Student–Newman–Keuls test (*p* < 0.05).

Extraction of POL from WE gave significantly different yields (*p* < 0.0001), being 13.4% for *Jania* (JAN), 40.6% for *Ecklonia* (ECK), and 58.7% for *Anabaena* sp. (AN) (data not shown). The effect of POL different concentrations on the colony growth rate of *B. cinerea* is reported in Figure 1. Two-way ANOVA indicated that POL and POL concentration factors and their interaction were significant (*p* < 0.05). *Anabaena* sp. POL at 0.5, 2.0, and 3.5 mg/mL significantly reduced colony growth rate by 20.3%, 32.5%, and 34.4%, respectively compared with the untreated control (Figure S1a). POL concentrations of 2.0 and 3.5 mg/mL reduced colony growth rate similarly and more than 0.5 mg/mL. *Ecklonia* sp. POL concentrations at 0.5, 2.0, and 3.5 mg/mL significantly reduced the colony growth rate in a similar way by an average of 22.5% compared with the untreated control. *Jania* sp. POL did not reduce *B. cinerea* colony growth rate. Among concentrations, at 2.0 and 3.5 mg/mL, AN POL showed a similar colony growth rate reduction to ECK POL.

Concerning the effect of POL on spore germination, CFUs and colony growth derived from CFUs of *B. cinerea,* the EC_50_ values for AN POL, ECK POL, and JAN POL were calculated (Table 2). Spore germination reduction ranged from 0.058 (0.027–0.127) mg/mL to 0.202 (0.118–0.346) mg/mL, but on the basis of 95% confidence limits overlap, differences were not significant (Figure S1b,c). No differences in EC_50_ values were found between AN POL and ECK POL both for CFUs and colony growth. When *B. cinerea* spores were exposed to JAN POL at 0.5, 2.0, and 3.5 mg/mL, both CFUs and CFU growth values were similar to those of untreated control. Values of CFUs of *B. cinerea* spores of untreated control was 86.0 ± 4.3% and varied from 82.5 ± 16.3% to 88.0 ± 9.6% for spores treated with JAN POL.

### 2.2. Effect of POL against B. cinerea on Strawberry Fruits

**Figure 2:**
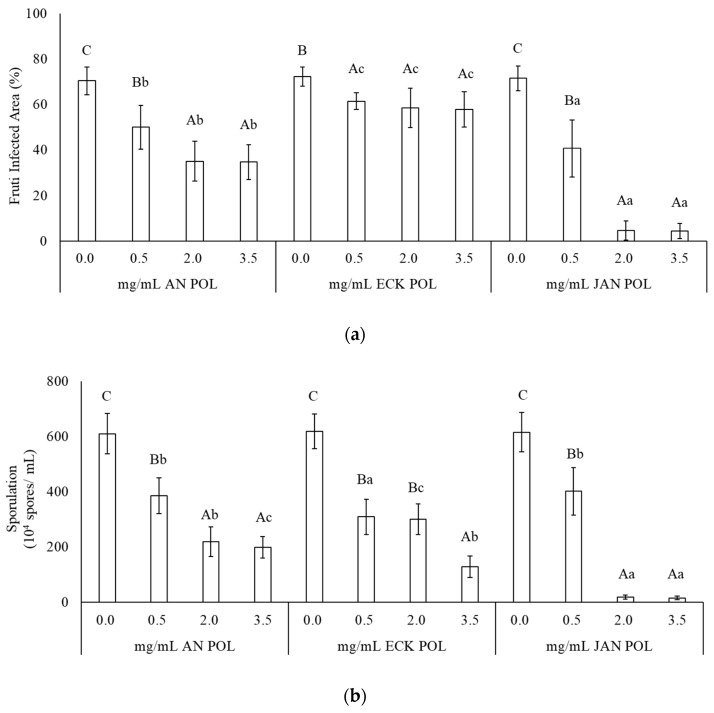
Infected area of strawberry fruit caused by *Botrytis cinerea* (**a**) and its sporulation (**b**) after pre-harvest treatment with different concentrations of polysaccharides from *Anabaena* sp. (AN), *Ecklonia* sp. (ECK), and *Jania* sp. (JAN). Polysaccharides and concentration factors and their interaction are significant, according to factorial ANOVA. (**a**) F_(2,240)_ = 270.0, *p* < 0.0001 (for treatment factor), F_(3,240)_ = 266.3, *p* < 0.0001 (for dose factor), F_(6,240)_ = 45.0, *p* < 0.05 (for interaction). (**b**) F_(2,96)_ = 23.0, *p* < 0.0001 (for treatment factor), F_(3,96)_ = 370.0, *p* < 0.0001 (for dose factor), F_(6,96)_ = 18.5, *p* < 0.05 (for interaction). Columns are mean values ± SD. The same uppercase letter within each POL treatment and the same lowercase letter within each concentration indicates no significant differences according to Student–Newman–Keuls test (*p* < 0.05).

The effect of treatment with POL from *Anabaena* sp., *Ecklonia* sp., and *Jania* sp. applied at different concentrations on strawberry fruits against *B. cinerea* is showed in Figure 2. Two-way ANOVA indicated that POL and POL concentration factors and their interaction were significant (*p* < 0.05). All polysaccharides applied at 0.5, 2.0, and 3.5 mg/mL significantly reduced fruit infected area compared to the untreated control (Figure 2a; Figure S2a–c). ECK POL inhibitory effect was lower compared to other POL and no differences were detectable among the different concentration. On the other hand, AN and JAN POL reduced the fruit infected area to a higher extent and in a dose-dependent manner. Among the concentrations, at 0.5, 2.0, and 3.5 mg/mL JAN POL showed the highest fruit infected area reduction (Figure 2a).

The effect of treatment with POL from AN, ECK, and JAN on *B. cinerea* sporulation on treated-infected fruit was then evaluated, as a measure of disease severity (Figure 2b). Two-way ANOVA indicated that POL and POL concentration factors and their interaction were significant (*p* < 0.05). All polysaccharides applied at all concentrations significantly reduced pathogen sporulation compared to the untreated control; however, substantial differences were seen among the different POL: similar to the effect of the fruit infected area (Figure 2a), AN and JAN reduced the sporulation capacity of *B. cinerea* grown on fruits up to 2 mg/mL concentration. However, JAN was much more effective, reducing sporulation ability up to 97.4%. POL from ECK inhibited *B. cinerea* sporulation of about 50% similarly at concentrations of 0.5 and 2.0 mg/mL. A higher inhibitory effect (79.2%) was detected at higher concentration (3.5 mg/mL). Among the concentrations, JAN POL at 2.0 and 3.5 mg/mL and ECK POL at 0.5 mg/mL showed the highest sporulation reduction.

## 3. Discussion

The use of algal polysaccharides for plant disease control as an alternative to synthetic products has been widely explored [19,28,29]. Indeed, green, brown, and red seaweeds contain several cell wall and storage polysaccharides such as ulvans, alginates, fucans, laminarin, and carrageenans acting as pathogen-associated molecular patterns and capable of inducing plant resistance [19]. They can stimulate rapid cellular changes associated with pathogen perception and defense activation, such as calcium concentration, oxidative burst, salicylic/jasmonic acids, ethylene signaling pathways, and the expression of pathogenesis-related proteins genes [19,30,31]. Polysaccharides from the brown alga *Ecklonia* sp. and from the red one *Jania* sp. as well as those extracted from the cyanobacterium *Anabaena* sp. have never been explored as resistance inducers for possible use in plant disease control. On the contrary, water extracts from *Ecklonia* sp. and *Anabaena* sp. have already shown antifungal activity against *Podosphaera xanthii* on zucchini as well as a water extract from *Corallina* sp., a red algae belonging to the same family of *Jania* sp. [9,32]. In particular, the water extract from *Anabaena* sp. increased defense-related enzymes in zucchini [9].

This study demonstrated that water extracts from *Ecklonia* sp., *Jania* sp., and *Anabaena* sp. exerted antifungal activity against *B. cinerea* growth. Antifungal activity of extracts from algae against fungal growth is widely reported [8,14,32,33,34]. De Corato et al. [14] obtained a high reduction of *B. cinerea* growth and spore germination with crude and hexane extracts from the brown algae *Laminaria digitate* and *Undaria pinnatifida* and from the red one *Porphyra umbilicalis*. Consistently, we report here that the extract from the brown alga *Ecklonia* sp. and red alga *Jania* sp. are active against *B. cinerea*. Extracts from the brown algae *Sargassum* spp. were effective against other fungal species, such as *Fusarium solani*, *Rhizoctonia solani*, *Aspergillus* spp., *Fusarium oxysporum*, and *Penicillium* spp. [33,34,35]. The antifungal activity of brown and red algae has been linked to several substances such as phenol and terpenes, as showed by [25] against *B*. *cinerea* and by [36] against a human pathogen.

With respect to cyanobacteria, they are a source of bioactive compounds, studied for their antifungal activity mainly against human fungal pathogens, such as the phenols and cyclic peptides active against *Candida albicans* [37]. Some studies have also reported their antifungal activity against fungal plant pathogens. Extracts from *Anabaena laxa* were active against *Aspergillus oryzae* and *Penicillium notatum* [38,39], whereas extracts from *Anabaena* species against *Pythium* sp., *Fusarium* sp., and *Rhizoctonia* sp. [40,41,42,43]. However, unlike our extraction method, in these studies, the extracts were obtained using organic solvents.

To our knowledge, no studies were published on the antifungal activity of polysaccharides extracted from algae and cyanobacteria against fungal plant pathogens. Considering that POL are abundant in algae and cyanobacteria, and that they are among the most active compounds, our study aimed to investigate the effects of these components extracted from *Anabaena* sp., *Ecklonia* sp., and *Jania* sp. against *B. cinerea* growth and strawberry grey mold by their application in pre- or post-harvest. We obtained different yields of POL from WE. *Anabaena* sp. showed the highest POL content since it is a cyanobacterium. Although cyanobacteria have an overall gram-negative structure, their peptidoglycan layer is considerably thicker than that of most gram-negative bacteria [44]. The different POL yields obtained from *Jania* sp. and *Ecklonia* sp. are consistent with the higher carbohydrate content in the brown alga *Chnoospora minima* than in *Jania adhaerens* [45]. Indeed, *Jania* sp. is characterized by a hard, calcareous skeleton mainly composed by calcite, which is typical of *Corallinaceae* [46]. Polysaccharides from *Anabaena* sp. and *Ecklonia* sp. were effective in reducing *B. cinerea* growth, while those from *Jania* sp. were not effective. In particular. for *Anabaena* sp., the antifungal activity depended on polysaccharide concentration and was more effective at 2.0 and 3.5 mg/mL. Polysaccharides from the brown seaweeds *Colpomenia sinuosa* and *Sargassum polycystum* showed antifungal activity against the human pathogen *Candida albicans* [47] and those from *Sargassum latifolium* against human viruses [48]. Skalicka-Woźniak et al. [49] found that polysaccharides extracted from the mushroom *Ganoderma lucidum* were also effective against gram-negative and gram-positive bacteria pathogenic for human at concentrations ranging from 0.075 to 5 mg/mL. These results are in line with those obtained with *Anabaena* sp. and *Ecklonia* sp. Polysaccharides, which showed antifungal activity against *B. cinerea* at a similar range of 0.5–3.5 mg/mL. *Anabaena* sp. and *Ecklonia* sp. polysaccharides also showed EC_50_ range values of CFUs (0.553 and 1.201 mg/mL, respectively) and CFU growth (1.064 and 2.087 mg/mL, respectively), which were comparable to MIC values of polysaccharides from *G. lucidum* (0.63–2.5 mg/mL) against several bacterial strains [49] and from polysaccharide gel extracted from fruit-hulls of durian against *Staphylococcus aureus* growth (0.64 mg/mL) [50]. The Sphagnum Moss has been studied as a polysaccharide source against fungi and bacteria in archaeological conservation. *Aspergillus* species, *E. coli* and *P. aeruginosa* (both gram-negative) were highly inhibited, while *Staphylococcus aureus* (gram-positive) was shown to be insensitive to *Sphagnum palustre* polysaccharides [51]. Several studies demonstrated that sulfated POL possesses several bioactive properties such as antimicrobial activity [20,21,22]. The absence of antifungal activity of *Jania* sp. POL against *B. cinerea* mycelium can be related to their structure molecular size and degree of sulfatation in the extracted POL. Polysaccharides extracted from *Anabaena* sp. and *Ecklonia* sp. and also those from *Jania* sp. strongly inhibited *B. cinerea* spore germination after 6 h exposure, the same amount of time necessary for *S. palustre* polysaccharides to kill 10,000 *Aspergillus* spp. spores [51], suggesting a similar inhibitory action mechanism. Spores were more sensitive than mycelium to POL as observed for chitosan against several plant pathogenic fungi [52].

Polysaccharides from *Anabaena* sp., *Ecklonia* sp., and *Jania* sp. reduced *B. cinerea* infection also on strawberry fruits, particularly if they were applied as a pre-harvest treatment. We suppose that disease symptoms reduction in case of polysaccharides from *Anabaena* sp. and *Ecklonia* sp. is due both to their direct effect on pathogen development, as observed in vitro, and to the activation of plant defense response. In particular, a plant defense activation mechanism can play a role in the effect of *Jania* sp. polysaccharides applied on strawberry fruit against the grey mold causal agent, considering that the pathogen inoculation was carried out 3 h after treatment and that *Jania* sp. polysaccharides displayed only a transient inhibitory effect on the fungal spore germination in vitro. Notably, on fruits, *Jania* sp. polysaccharides showed the highest efficacy against grey mold disease and pathogen sporulation compared to *Ecklonia* sp. and *Anabaena* sp.

The elicitation of plant defense response by algal polysaccharides is well-known. Activating signaling pathways, among which salicylic acid, jasmonic acid, and ethylene either alone or in combination, play major roles in local and systemic induction of defense responses [53,54,55,56]. Polysaccharides and derived oligosaccharides extracted from algae, such as agarans and carrageenans in red algae and alginates, fucans, and laminarin in brown ones, can increase the expression of defense genes and enzymes such as chitinase and glucanase, involved in plant defense response. Laminarin from the brown alga *Laminaria digitata* induced the release of hydrogen peroxide in tobacco cells and a transient increase in phenylalanine ammonia lyase activity with a maximal level at 4 h, a sustained increase in lipoxygenase activity up to 20 h and the accumulation of PR-1, PR-2 (glucanase), PR-3 (chitinase), and PR-5 at 48 h of treatment. These enzymatic activities are probably involved in their ability to control the infection caused by *Erwinia carotovora* [57]. The application of laminarin and alginate reduced the development of wilt symptoms caused by *Verticillium dahliae* on olive twigs, stimulating the phenolic metabolism [58]. Moreover, alginates, reduced the pathogen growth in vitro.

Concerning red algae polysaccharides, carrageenans induced protection against a broad range of pathogens such as tobacco mosaic virus (TMV), *B*. *cinerea* and *E. carotovora* on tobacco [19]. Again on tobacco, [59] showed that carrageenan infiltrated in leaves increased the expression of genes coding for a sesquiterpene cyclase involved in the synthesis of the antimicrobial terpenoid capsidiol, of PR-3 proteins (basic chitinases) and proteinase inhibitor with antipathogenic activity. Carrageenans were also able to strengthen tobacco cell walls and induce the production of phenolic compounds leading to the reduction of the number of TMV spots [60].

Post-harvest treatment of detached fruits was not effective against grey mold, despite all polysaccharides being active against *B. cinerea* spore germination and development in vitro. This supports the hypothesis that the inhibitory action of these algae and cyanobacteria POL is displayed more through a resistance induction mechanism on the fruit still attached to the plant than through direct antimicrobial activity. Resistance induction implies a more active fruit metabolic activity, possibly quickly decreasing upon fruit detachment [61,62,63].

To our knowledge, the exploitation of cyanobacteria polysaccharide against plant pathogens is not known, despite their very abundant presence as a mucilaginous external layer around the cyanobacterial cell. Our work showed for the first time that cyanobacteria are a source of polysaccharides that have the potential to act as plant protectant compounds both directly through antifungal activity or indirectly as resistance inducers.

In conclusion, this study shows that polysaccharides extracted from *Anabaena* sp., *Ecklonia* sp., and *Jania* sp. are active both against *B. cinerea* development and grey mold disease in pre-harvest treatment. Once these effects will be confirmed in large scale experiments, these components may provide an effective protection tool useful in environmentally-friendly disease management to reduce the impact of hazardous pesticides.

## 4. Materials and Methods

### 4.1. Preparation of Water Extract, WE, Extraction of Polysaccharides, POL, and Pathogen

Lyophilized biomass of *Anabaena* sp. BEA 0300B (AN) and dry thallus of *Ecklonia* sp. (ECK) and *Jania* sp. (JAN) were provided by the Spanish Bank of Algae (BEA), University of Las Palmas de Gran Canaria. Dry thallus of ECK and JAN was ground to a fine powder with mortar and pestle. Water extracts (WE) from AN, ECK, and JAN were obtained by suspending each powder in sterile distilled water (0.5%) under continuous stirring at 50 °C for 12 h and then filtered [9]. Polysaccharides were extracted from WE [64] and selectively precipitated with 2% (w/v) *O*-*N*-cetylpyridinium bromide (Cetavlon) [65]. Then, they were purified with 4 M NaCl, flocculated with 96% (v/v) ethanol and centrifuged (10,000 *g* for 10 min; Beckman Coulter Avanti J-26 XP, Indianapolis, IN, USA). Polysaccharides were dialyzed (Membra-Cel MD34 14 × 100 CLR, Viskase Corporation, Chicago, IL, USA, molecular weight cut-off 14,000 daltons) against 2 M NaCl, and lyophilized. The yield related to the POL biomass was calculated [66] with modifications: (solubilized (obtained)/biomass (provided)) × 100. Solubilized (obtained) is the lyophilized polysaccharides biomass (g) obtained from water extract and biomass (provided) is the biomass (g) used to make the water extract.

A monoconidial culture of *B. cinerea* derived from the CRIOF (Centro per la Conservazione e Trasformazione dei Prodotti Ortofrutticoli, University of Bologna) collection was used. The fungus pathogenicity was verified through the inoculation of spore suspension on strawberry fruits and waiting for the symptom appearance. The spore suspension was obtained from 7-day-old colonies grown on potato dextrose agar (PDA 3.9%, Difco, Detroit, MI, USA) at 24 °C. For the experiments, portions of *B. cinerea* colony were transferred on PDA supplemented with 60 mg of streptomycin sulfate (Sigma-Aldrich Co., Saint Louis, MO, USA), and incubated at 24 °C under natural light conditions for seven days.

### 4.2. Antifungal Activity of WE on B. cinerea Colony Growth

The effect of different concentrations of WE on *B. cinerea* colony growth was evaluated on PDA medium in Petri dish 90 mm diameter. The WE concentration range, dilution factor 1:2, was chosen considering the standard dose (DS) equal to 1 and varied from 2-fold the DS (2DS) to 0.5DS. The SD of WE was 5 mg/mL [32]. Fungal portions of 7 mm diameter were cut from the 10-day-old colony and transferred in test-tube containing a 600 µL aliquot of each WE concentration. After 6 h, colony portions were placed on PDA medium and incubated at 24 °C in the dark. Colony growth was measured daily in two directions, along two mutually perpendicular diameters until the growth of the untreated control reached 80 mm diameter. Three dishes were considered for each treatment and for water control. The experiment was repeated twice.

### 4.3. Antifungal Activity of POL on B. cinerea Colony Growth, Spore Germination, and Colony Forming Units, CFUs

To evaluate the effects of POL on fungal colony growth, spore germination, and CFUs, three POL concentrations were used, 0.5, 2.0, and 3.5 mg/mL. To study the effect on *B*. *cinerea* colony growth, portions of 7 mm diameter were cut from the 10-day-old colony and transferred in test-tube containing a 600 µL aliquot of each POL concentration for 6 h. Colony portions were then placed on a PDA medium in a Petri dish and incubated at 24 °C for 4 d in the dark. Colony growth was measured daily in two directions, along two mutually perpendicular diameters. Three dishes were considered for each treatment and for water control. The experiment was repeated twice.

The effect of POL on spore germination and on CFUs were performed in flasks containing a mixture of each POL concentration with *B. cinerea* spore suspension 5 × 10^2^ spores/mL. The control consisted of sterile distilled water added to a *B. cinerea* spore suspension. Flasks were manually stirred for 10 sec. and incubated at 24 °C for 4 h in the dark. For spore germination, four 50 µL-volume drops from each flask were put on microscope glass slides and observed at the optical microscope (×400 magnification). Germinated spores were counted in a total of 100 spores. To evaluate the antifungal effect of POL on *B. cinerea* CFUs, 50 µL from each flask was gently spread on the surface of a PDA medium in a Petri dish. Four Petri dishes were used for each treatment and for the untreated control. Petri dishes were incubated at 24 °C in the dark. After 24 h of incubation, the CFU number was counted in treated and untreated dishes. After 48 h of incubation, the diameter of five colonies per dish derived from CFUs were measured along two mutually perpendicular diameters. The experiment was repeated twice.

### 4.4. Effect of Polysaccharides against B. cinerea on Strawberry Fruits

Strawberry plants cv. Cristal were transplanted in 30 cm diameter pots filled with peat substrate, and maintained in a greenhouse under natural light conditions, at BEA (Banco Español de Algas), Muelle de Taliarte, Gran Canaria, Spain. Plants were irrigated and fertilized regularly (N:P:K, 12:12:17).

About twenty days after blossom, ripened fruits were used for two experiments in order to verify the effect of pre-harvest or post-harvest treatment with POL from AN, ECK, and JAN against *B. cinerea*. In the pre-harvest treatment, twenty fruits still attached to the plants were immerged in polysaccharides solutions for 2 min. After 3 h, treated fruits were harvested and inoculated by spraying 0.5 mL *B. cinerea* spore suspension (1 × 10^5^ spores/mL). In the post-harvest treatment, twenty fruits were harvested, and then treated by immersion in POL suspensions for 2 min. After 3 h, treated fruits were inoculated as above described.

In both cases, two controls were considered: fruits treated with water without fungus inoculation, and fruits treated with water followed by pathogen inoculation.

Disease symptoms were evaluated as a percentage of fruit area showing symptoms of grey mold (infected area) over the total area inoculated and as fruit sporulation. The experiment was repeated twice.

## Figures and Tables

**Table 1:** Effect of different water extract (WE) concentrations of *Anabaena* sp. (AN), *Ecklonia* (ECK), and *Jania* (JAN) on *Botrytis cinerea* colony growth rate.

Concentration (mg/mL) WE Treatment	0.0	2.5	5.0	10.0	Mean
	Colony Growth Rate (mm/day)				
AN	18.3 ± 0.6	16.3 ± 0.3	16.0 ± 1.3	16.2 ± 0.3	16.7 ± 1.2 A
ECK	18.2 ± 0.8	18.2 ± 0.3	16.8 ± 0.8	16.5 ± 0.5	17.4 ± 0.9 A
JAN	18.7 ± 0.6	17.5 ± 0.5	16.8 ± 0.3	16.8 ± 0.8	17.5 ± 0.9 A
Mean	18.4 ± 0.6 c	17.3 ± 0.9 b	16.6 ± 0.9 a	16.5 ± 0.6 a	

Treatment and dose factors are significant, whereas their interaction is not significant according to factorial ANOVA. F_(2,36)_ = 5.1, *p* < 0.0001 (for treatment factor), F_(3,36)_ = 16.8, *p* < 0.0001 (for dose factor). Mean values ± SD followed by the same uppercase letter in a row and by the same lowercase letter in a column are not significantly different according to Student–Newman–Keuls test (*p* < 0.05).

**Table 2:** Effective concentrations (EC_50_) and 95% confidence limits of polysaccharides (POL) from *Anabaena* sp. (AN), *Ecklonia* sp. (ECK), and *Jania* sp. (JAN) required to reduce by 50% spore germination, colony forming units (CFUs), and CFU colony growth of *Botrytis cinerea*.

Treatment	EC_50_ POL (mg/mL)		
	Spore Germination	CFUs	CFU Colony Growth
AN POL	0.058 (0.027–0.127)	0.553 (0.255–1.202)	1.064 (0.665–1.703)
ECK POL	0.096 (0.048–0.195)	1.201 (0.584–5.392)	2.087 (1.627–2.676)
JAN POL	0.202 (0.118–0.346)	nd ^a^	nd ^a^

^a^ not detectable, not different from the untreated control.

## Supplementary Materials

The following are available online at https://www.mdpi.com/1660-3397/17/5/299/s1, Figure S1: Effect of different concentrations of polysaccharides from *Anabaena* sp. on *Botrytis cinerea* colony growth 3 days after treatment (**a**) and spore germination (**b**). Untreated spore (**c**); Figure S2. Effect of preharvest treatment with polysaccharides (POL) from *Jania* sp. (JAN) on grey mold control of strawberry fruits six days after treatment. Untreated fruits (**a**); fruits treated with JAN-POL at 2.0 mg/mL (**b**) and fruits treated with JAN-POL at 3.5 mg/mL (**c**).

## References

[1] Dallagnol L.J., Ferreira L.V., Araujo-Filho J.A., Camargo L.E.A., de Castro-Moretti F.R. (2013). Gray mold caused by *Botryotinia fuckeliana* on edible pods of pea in Brazil. Plant Dis..

[2] Michailides T.J., Elmer P.A.G. (2007). *Botrytis* gray mold of kiwifruit caused by *Botrytis cinerea* in the United States and New Zealand. Plant Dis..

[3] Ciliberti N., Fermaud M., Roudet J., Rossi V. (2015). Environmental conditions affect *Botrytis cinerea* infection of mature grape berries more than the strain or transposon genotype. Phytopathology.

[4] Erper I., Celik H., Turkkan M., Cebi Kilicoglu M. (2015). First report of *Botrytis cinerea* on golden berry. Australas. Plant Dis. Notes.

[5] Rodríguez A., Acosta A., Rodríguez C. (2014). Fungicide resistance of *Botrytis cinerea* in tomato greenhouses in the Canary Islands and effectiveness of non-chemical treatments against gray mold. World J. Microbiol. Biotechnol..

[6] Blanco C., De Los Santos B., Romero F. (2006). Relationship between concentrations of *Botrytis cinerea* conidia in air, environmental conditions, and the incidence of grey mould in strawberry flowers and fruits. Eur. J. Plant Pathol..

[7] Rizvi M.A., Shameel M. (2004). Studies on the bioactivity and elementology of marine algae from the coast of Karachi, Pakistan. Phyther. Res..

[8] Righini H., Roberti R., Baraldi E. (2018). Use of algae in strawberry management. J. Appl. Phycol..

[9] Roberti R., Galletti S., Burzi P.L., Righini H., Cetrullo S., Perez C. (2015). Induction of defense responses in zucchini (*Cucurbita pepo*) by *Anabaena* sp. water extract. Biol. Control.

[10] Sivakumar S.R. (2014). Antibacterial potential of white crystalline solid from red algae *Porteiria hornemanii* against the plant pathogenic bacteria. African, J. Agric. Res..

[11] Jiménez E., Dorta F., Medina C., Ramírez A., Ramírez I., Peña-Cortés H. (2011). Anti-phytopathogenic activities of macro-algae extracts. Mar. Drugs.

[12] Arunkumar K., Sivakumar S.R., Rengasamy R. (2010). Review on bioactive potential in seaweeds (Marine Macroalgae): A special emphasis on bioactivity of seaweeds against plant pathogens. Asian J. Plant Sci..

[13] Kumar C.S., Sarada D.V.L., Rengasamy R. (2008). Seaweed extracts control the leaf spot disease of the medicinal plant *Gymnema sylvestre*. Indian J. Sci. Technol..

[14] De Corato U., Salimbeni R., De Pretis A., Avella N., Patruno G. (2017). Antifungal activity of crude extracts from brown and red seaweeds by a supercritical carbon dioxide technique against fruit postharvest fungal diseases. Postharvest Biol. Technol..

[15] Esserti S., Smaili A., Rifai L.A., Koussa T., Makroum K., Belfaiza M., Kabil E.M., Faize L., Burgos L., Alburquerque N. (2017). Protective effect of three brown seaweed extracts against fungal and bacterial diseases of tomato. J. Appl. Phycol..

[16] Jayaraman J., Norrie J., Punja Z.K. (2011). Commercial extract from the brown seaweed *Ascophyllum nodosum* reduces fungal diseases in greenhouse cucumber. J. Appl. Phycol..

[17] Agarwal P., Patel K., Das A.K., Ghosh A., Agarwal P.K. (2016). Insights into the role of seaweed *Kappaphycus alvarezii* sap towards phytohormone signalling and regulating defense responsive genes in *Lycopersicon esculentum*. J. Appl. Phycol..

[18] Jaki B., Zerbe O., Heilmann J., Sticher O. (2001). Two novel cyclic peptides with antifungal activity from the cyanobacterium *Tolypothrix byssoidea* (EAWAG 195). J. Nat. Prod..

[19] Vera J., Castro J., Gonzalez A., Moenne A. (2011). Seaweed polysaccharides and derived oligosaccharides stimulate defense responses and protection against pathogens in plants. Mar. Drugs.

[20] Pierre G., Sopena V., Juin C., Mastouri A., Graber M., Maugard T. (2011). Antibacterial activity of a sulfated galactan extracted from the marine alga *Chaetomorpha aerea* against *Staphylococcus aureus*. Biotechnol. Bioprocess Eng..

[21] Marudhupandi T., Thipparamalai Thangappan Ajith K. (2013). Antibacterial effect of fucoidan from *Sargassum wightii* against the chosen human bacterial pathogens. Int. Curr. Pharm. J..

[22] Jun J.-Y., Jung M.-J., Jeong I.-H., Yamazaki K., Kawai Y., Kim B.-M. (2018). Antimicrobial and antibiofilm activities of sulfated polysaccharides from marine algae against dental plaque bacteria. Mar. Drugs.

[23] Pérez M.J., Falqué E., Domínguez H. (2016). Antimicrobial action of compounds from marine seaweed. Mar. Drugs.

[24] Rioux L.E., Turgeon S.L., Beaulieu M. (2007). Characterization of polysaccharides extracted from brown seaweeds. Carbohydr. Polym..

[25] Liu M., Wang G., Xiao L., Xu A., Liu X., Xu P., Lin X. (2014). Bis(2,3-dibromo-4,5-dihydroxybenzyl) ether, a marine algae derived bromophenol, inhibits the growth of *Botrytis cinerea* and interacts with DNA molecules. Mar. Drugs.

[26] Pugh N., Samir A.R., Hala N.E., Mahmoud A.E., David S.P. (2001). Isolation of three high molecular weight polysaccharide preparations with potent immunostimulatory activity from *Spirulina platensis*, *Aphanizomenon flos*-*aquae* and *Chlorella pyrenoidosa*. Planta Med..

[27] Hayashi K., Hayashi T., Kojima I. (1996). A natural sulfated polysaccharide, calcium spirulan, isolated from *Spirulina platensis*: In vitro and ex vivo evaluation of anti-herpes simplex virus and anti-human immunodeficiency virus activities. AIDS Res. Hum. Retroviruses.

[28] Hahn M.G., Darvill A.G., Albersheim P. (2008). Host-Pathogen Interactions: XIX. the endogenous elicitor, a fragment of a plant cell wall polysaccharide that elicits phytoalexin accumulation in soybeans. Plant Physiol..

[29] Stadnik M.J., Freitas M.B. (2014). de Algal polysaccharides as source of plant resistance inducers. Trop. Plant Pathol..

[30] Jaulneau V., Lafitte C., Jacquet C., Fournier S., Salamagne S., Briand X., Esquerré-Tugayé M.-T., Dumas B. (2010). Ulvan, a sulfated polysaccharide from green algae, activates plant immunity through the jasmonic acid signaling pathway. J. Biomed. Biotechnol..

[31] Zhao G., Zhao J., Peng L., Zou L., Wang J., Zhong L., Xiang D. (2012). Effects of yeast polysaccharide on growth and flavonoid accumulation in *Fagopyrum tataricum* sprout cultures. Molecules.

[32] Roberti R., Righini H., Reyes C.P., Roberti R., Righini H., Reyes C.P. (2016). Activity of seaweed and cyanobacteria water extracts against *Podosphaera xanthii* on zucchini. Ital. J. Mycol..

[33] Khallil A.M., Daghman I.M., Fady A.A. (2015). Antifungal potential in crude extracts of five selected brown seaweeds collected from the western Libya coast. J. Microbiol. Mod. Tech..

[34] Ibraheem I.B., Hamed S.M., Abd elrhman A.A., Mohamed Farag F., Abdel-Raouf N. (2017). Antimicrobial activities of some brown macroalgae against some soil borne plant pathogens and in vivo management of *Solanum melongena* root diseases. Aust. J. Basic Appl. Sci. J. Basic Appl. Sci..

[35] Mabrouk S.S., El-Shayeb N.M.A., El-Refai A.H., Sallam L.A.R., Hamdy A.A. (1985). Inhibitory activities of some marine algae on aflatoxin accumulation. Appl. Microbiol. Biotechnol..

[36] Eom S.H., Kim Y.M., Kim S.K. (2012). Antimicrobial effect of phlorotannins from marine brown algae. Food Chem. Toxicol..

[37] De Cano M.M.S., de Mulé M.C., de Caire G.Z., de Halperin D.R. (1990). Inhibition of *Candida albicans* and *Staphylococcus aureus* by phenolic compounds from the terrestrial cyanobacterium *Nostoc muscorum*. J. Appl. Phycol..

[38] Frankmölle W.P., Larsen L.K., Caplan F.R., Patterson G.M.L., Knubel G., Levine I.A., Moore R.E. (1992). Blue-green alga *Anabaena laxa*. I. Isolation and biological properties. J. Antibiot. (Tokyo).

[39] Frankmölle W.P., Knubel G., Moore R.E., Patterson G.M.L. (1992). Blue-green alga *Anabaena laxa*. II. Structures of laxaphycins a,b, d, and e. J. Antibiot. (Tokyo).

[40] Moon S.S., Lu Chen J., Moore R.E., Patterson G.M.L. (1992). Calophycin, a fungicidal cyclic decapeptide from the terrestrial blue-green alga *Calothrix fusca*. J. Org. Chem..

[41] Prasanna R., Nain L., Tripathi R., Gupta V., Chaudhary V., Middha S., Joshi M., Ancha R., Kaushik B.D. (2008). Evaluation of fungicidal activity of extracellular filtrates of cyanobacteria—Possible role of hydrolytic enzymes. J. Basic Microbiol..

[42] Radhakrishnan B., Prasanna R., Jaiswal P., Nayak S., Dureja P. (2009). Modulation of biocidal activity of *Calothrix* sp. and *Anabaena* sp. by environmental factors. Biologia (Bratisl).

[43] Manjunath M., Prasanna R., Nain L., Dureja P., Singh R., Kumar A., Jaggi S., Kaushik B.D. (2010). Biocontrol potential of cyanobacterial metabolites against damping off disease caused by *Pythium aphanidermatum* in solanaceous vegetables. Arch. Phytopathol. Plant Prot..

[44] Hoiczyk E., Hansel A. (2000). Cyanobacterial cell walls: News from an unusual prokaryotic envelope. J. Bacteriol..

[45] Shanura Fernando I., Asanka Sanjeewa K.K., Samarakoon K.W., Lee W.W., Kim H.S., Kim E.A., Gunasekara U.K.D.S.S., Abeytunga D.T.U., Nanayakkara C., de Silva E.D. (2017). FTIR characterization and antioxidant activity of water soluble crude polysaccharides of Sri Lankan marine algae. Algae.

[46] Johansen H.W., Taylor & Francis Group (2018). Coralline Algae, a First Synthesis. Coralline Algae, A First Synthesis.

[47] Kantachumpoo A., Chirapart A. (2010). Components and antimicrobial activity of polysaccharides extracted from thai brown seaweeds. Kasetsart, J. Nat. Sci..

[48] Asker M.M.S., Sahera F.M., Ali F.M., El-Sayed O.H. (2007). Chemical structure and antiviral activity of water-soluble sulfated polysaccharides from *Surgassum latifolium*. J. Appl. Sci. Res..

[49] Skalicka-Woźniak K., Szypowski J., Łoś R., Siwulski M., Sobieralski K., Głowniak K., Malm A. (2012). Evaluation of polysaccharides content in fruit bodies and their antimicrobial activity of four *Ganoderma lucidum* (W Curt.: Fr.) P. Karst. strains cultivated on different wood type substrates. Acta Soc. Bot. Pol..

[50] Lipipun V., Nantawanit N., Pongsamart S. (2002). Antimicrobial activity (*in vitro*) of polysaccharide gel from durian fruit-hulls. Songklanakarin, J. Sci. Tehcnol..

[51] Zaitseva N. (2009). A Polysaccharide Extracted from Sphagnum Moss as Antifungal Agent in Archaeological Conservation. Ph.D. Thesis.

[52] Palma-Guerrero J., Jansson H.B., Salinas J., Lopez-Llorca L.V. (2008). Effect of chitosan on hyphal growth and spore germination of plant pathogenic and biocontrol fungi. J. Appl. Microbiol..

[53] Hammond-Kosack K.E., Jones J.D.G. (1996). Resistance gene-dependent plant defense responses. Plant Cell.

[54] Reymond P., Farmer E.E. (1998). Jasmonate and salicylate as global signals for defense gene expression. Curr. Opin. Plant Biol..

[55] Paulert R., Talamini V., Cassolato J.E.F., Duarte M.E.R., Noseda M.D., Smania A., Stadnik M.J. (2009). Effects of sulfated polysaccharide and alcoholic extracts from green seaweed Ulva fasciata on anthracnose severity and growth of common bean (*Phaseolus vulgaris* L.). J. Plant Dis. Prot..

[56] Sharma H.S.S., Fleming C., Selby C., Rao J.R., Martin T. (2014). Plant biostimulants: A review on the processing of macroalgae and use of extracts for crop management to reduce abiotic and biotic stresses. J. Appl. Phycol..

[57] Klarzynski O., Plesse B., Joubert J.-M., Yvin J.-C., Kopp M., Kloareg B., Fritig B. (2000). Linear β-1,3 glucans are elicitors of defense responses in tobacco. Plant Physiol..

[58] Ben Salah I., Aghrouss S., Douira A., Aissam S., El Alaoui-Talibi Z., Filali-Maltouf A., El Modafar C. (2018). Seaweed polysaccharides as bio-elicitors of natural defenses in olive trees against *Verticillium* wilt of olive. J. Plant Interact..

[59] Mercier L., Lafitte C., Borderies G., Briand X., Esquerré-Tugayé M.T., Fournier J. (2001). The algal polysaccharide carrageenans can act as an elicitor of plant defense. New Phytol..

[60] Ghannam A., Abbas A., Alek H., Al-Waari Z., Al-Ktaifani M. (2013). Enhancement of local plant immunity against tobacco mosaic virus infection after treatment with sulphated-carrageenan from red alga (*Hypnea musciformis*). Physiol. Mol. Plant Pathol..

[61] Zheng Y., Sheng J., Zhao R., Zhang J., Lv S., Liu L., Shen L. (2011). Preharvest l -arginine treatment induced postharvest disease resistance to *Botrysis cinerea* in tomato fruits. J. Agric. Food Chem..

[62] Feliziani E., Landi L., Romanazzi G. (2015). Preharvest treatments with chitosan and other alternatives to conventional fungicides to control postharvest decay of strawberry. Carbohydr. Polym..

[63] Yao H., Tian S. (2005). Effects of pre- and post-harvest application of salicylic acid or methyl jasmonate on inducing disease resistance of sweet cherry fruit in storage. Postharvest Biol. Technol..

[64] Abdala Díaz R.T., Chabrillón M., Cabello-Pasini A., Gómez-Pinchetti J.L., Figueroa F.L. (2010). Characterization of polysaccharides from *Hypnea spinella* (Gigartinales) and *Halopithys incurva* (Ceramiales) and their effect on RAW 264.7 macrophage activity. J. Appl. Phycol..

[65] Morris Quevedo H.J., Martínez Manrique C., Abdala Díaz R., Cobas Pupo G. (2000). Evidencias preliminares de la actividad inmunomoduladora de la fracción polisacárida de origen marino Pc-1. Rev. Cubana Oncol..

[66] Álvarez-Gómez F., Korbee N., Figueroa F.L. (2016). Analysis of antioxidant capacity and bioactive compounds in marine macroalgal and lichenic extracts using different solvents and evaluation methods. Cienc. Mar..

