# Supplementary material for: Different Antifungal Activity of Anabaena sp., Ecklonia sp., and Jania sp. against Botrytis cinerea
Source: Mar Drugs. 2019 May 20;17(5):299. doi: 10.3390/md17050299 (PMC6562623; doi:10.3390/md17050299)
Supplement: Supplementary file 1 [file marinedrugs-17-00299-s001.pdf]

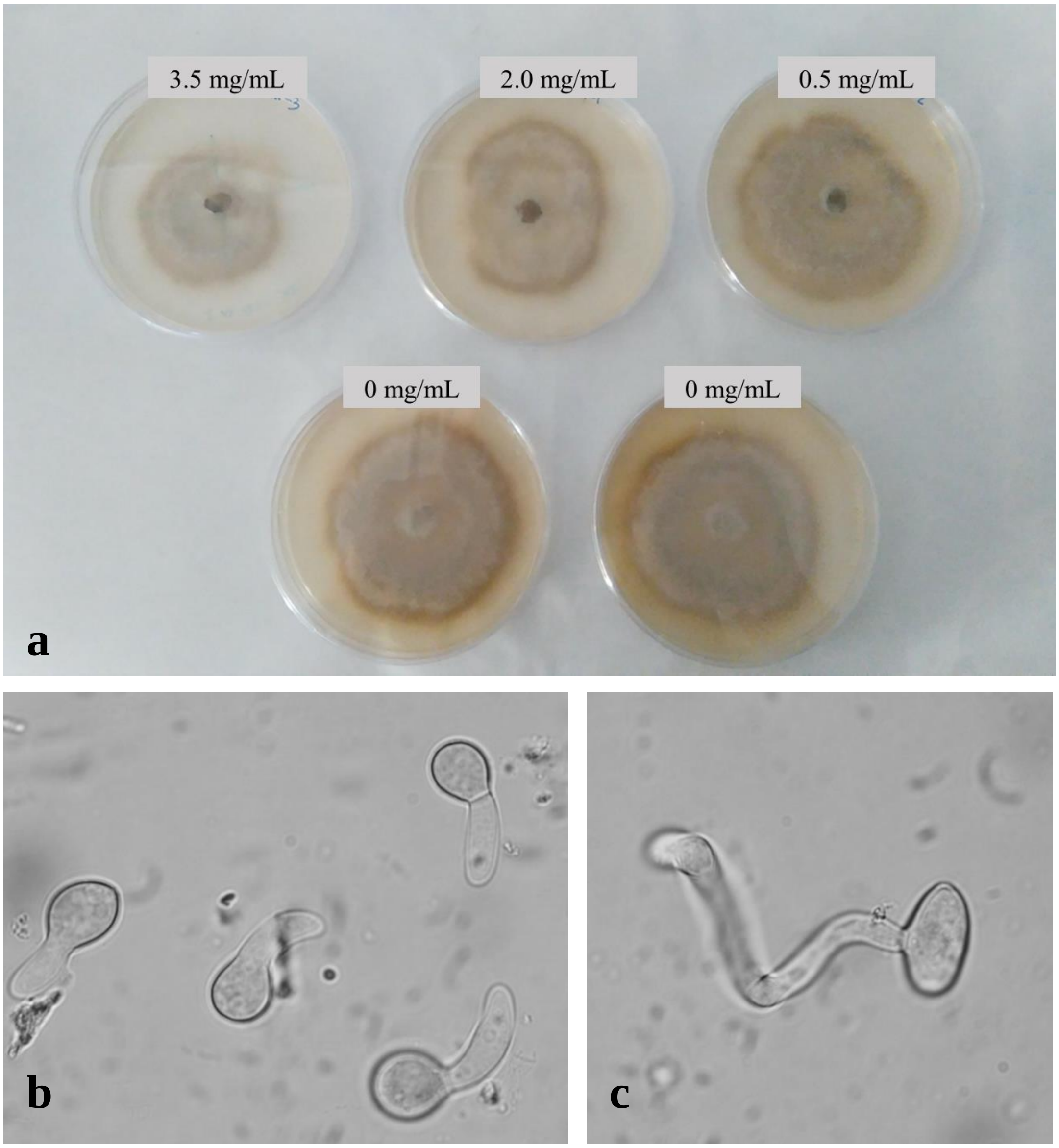

Figure S1. Effect of different concentrations of polysaccharides from *Anabaena* sp. on *Botrytis cinerea* colony growth 3 days after treatment (a) and spore germination (b). Untreated spore (c).

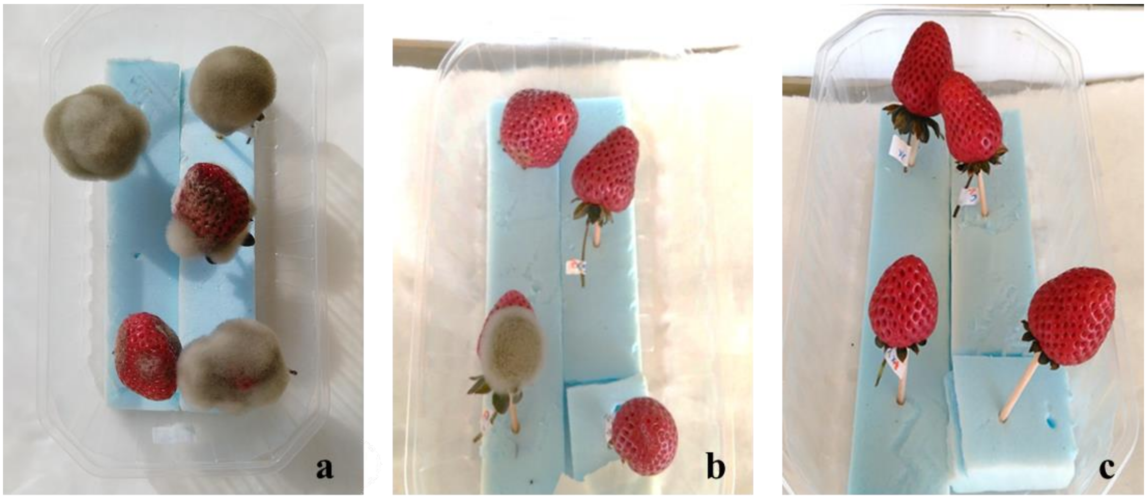

Figure S2. Effect of preharvest treatment with polysaccharides (POL) from *Jania* sp. (JAN) on grey mould control of strawberry fruits six days after treatment. Untreated fruits (a); fruits treated with JAN-POL at 2.0 mg/mL (b) and fruits treated with JAN-POL at 3.5 mg/mL (c).
